# Phantom-based evaluation of dose exposure of ultrafast combined kV-MV-CBCT towards clinical implementation for IGRT of lung cancer

**DOI:** 10.1371/journal.pone.0187710

**Published:** 2017-11-10

**Authors:** Anna Arns, Manuel Blessing, Jens Fleckenstein, Dzmitry Stsepankou, Judit Boda-Heggemann, Juergen Hesser, Frank Lohr, Frederik Wenz, Hansjoerg Wertz

**Affiliations:** 1 Department of Radiation Oncology, Universitaetsmedizin Mannheim, Medical Faculty Mannheim, Heidelberg University, Mannheim, Germany; 2 Struttura Complessa di Radioterapia, Dipartimento di Oncologia, Az. Ospedaliero-Universitaria di Modena, Modena, Italy; North Shore Long Island Jewish Health System, UNITED STATES

## Abstract

**Purpose:**

Combined ultrafast 90°+90° kV-MV-CBCT within single breath-hold of 15s has high clinical potential for accelerating imaging for lung cancer patients treated with deep inspiration breath-hold (DIBH). For clinical feasibility of kV-MV-CBCT, dose exposure has to be small compared to prescribed dose. In this study, kV-MV dose output is evaluated and compared to clinically-established kV-CBCT.

**Methods:**

Accurate dose calibration was performed for kV and MV energy; beam quality was determined. For direct comparison of MV and kV dose output, relative biological effectiveness (RBE) was considered. CT dose index (CTDI) was determined and measurements in various representative locations of an inhomogeneous thorax phantom were performed to simulate the patient situation.

**Results:**

A measured dose of 20.5mGE (Gray-equivalent) in the target region was comparable to kV-CBCT (31.2mGy for widely-used, and 9.1mGy for latest available preset), whereas kV-MV spared healthy tissue and reduced dose to 6.6mGE (30%) due to asymmetric dose distribution. The measured weighted CTDI of 12mGE for kV-MV lay in between both clinical presets.

**Conclusions:**

Dosimetric properties were in agreement with established imaging techniques, whereas exposure to healthy tissue was reduced. By reducing the imaging time to a single breath-hold of 15s, ultrafast combined kV-MV CBCT shortens patient time at the treatment couch and thus improves patient comfort. It is therefore usable for imaging of hypofractionated lung DIBH patients.

## Introduction

Image-guided radiotherapy (IGRT) enabled dose escalation to limited volumes and at the same time a reduction of normal tissue complications in external-beam-radiotherapy (EBRT) and stereotactic-body-radiotherapy (SBRT) [1,2]. The most commonly used technique for pre-treatment imaging is a perpendicularly mounted kilovoltage- (kV) Cone-Beam Computed Tomography (kV-CBCT) [3–5] while Megavoltage- (MV) treatment beam has only been used on a limited basis as MV-CBCT [6,7] due to reduced soft-tissue contrast and reduced quantum efficiency.

An approach increasingly employed to reduce for lung and liver motion is to treat the patient during computer-controlled breath-hold (deep-inspiratory breath-hold, DIBH) [8]. While the radiation treatment can now be performed within only a few breath-holds, CBCT image acquisition in repeated breath-hold takes about 3-4min. Although this “start/stop” CBCT imaging approach in combination with multiple breath-holds results in images of excellent image quality [9,10], it considerably extends the imaging time into the range of flattening filter free treatment delivery [11,12]. CBCT image acquisition within one single breath-hold (typically 10-20s) would result in high treatment precision with dramatically shortened overall treatment times while significantly increasing patient comfort.

Our novel imaging technique of combined ultrafast kV-MV-CBCT performs image acquisition within one breath-hold of 15s [13,14]. KV-MV-CBCT is based on the initial step-and-shoot technique introduced by Yin et al. [15], but was fully developed by us to be performed dynamically to be completed within 15s, which is a prerequisite for clinical implementation. KV-MV-CBCT is realized by simultaneous image acquisition of 90° kV and 90° MV projections, resulting in 180° image information (+ cone-angle). It can be operated on a clinically commissioned linear accelerator (Elekta Synergy linac) with minimal modifications [14]. The development of kV-MV-CBCT is finalized, the workflow is fully automated [16] and the image acquisition as well as image registration can be performed in real-time [17].

For the implementation of kV-MV-CBCT into clinical routine, imaging dose exposure to the patient has to be investigated and compared to clinically-established techniques. A standard dose value for a kV-MV-CBCT preset has to be defined for future quality assurance and documentation. We performed a comparison of imaging dose applied with kV-CBCT standard presets currently used in clinical routine. First, a lung tumor patient was simulated with an inhomogeneous thorax phantom and the dose exposure was measured at representative positions. Second, the weighted CT dose index was determined with an appropriate CT phantom. Proper ionization chamber calibration was performed for applied kV and MV energies, as well as the relative biological effectiveness was considered.

## Methods

KV-MV-CBCT imaging was developed on a clinical 6MV Elekta Synergy linac (Integrity v1.2), equipped with a foldout MV detector (iView GT v3.4) and a perpendicularly mounted kV-CBCT-system (XVI v4.2.2). To compare kV-MV-CBCT with clinically-established techniques, not only the Chest-preset of the Elekta Synergy linac was applied in the phantom dose measurements but also the Chest-preset of the latest available kV-CBCT software version (XVI v5.0.2), installed on a clinical Elekta VersaHD linac (all Elekta AB, Stockholm, Sweden).

### Setup of conventional kV-CBCT and kV-MV-CBCT

The imaging dose outputs of the following three imaging techniques were compared in this study (technical setups are summarized in Table 1):

- **Clinical kV-Chest**_**1**_**-CBCT**
  On the Elekta Synergy linac, the still widely-used XVI version 4.2.2 was installed. The preset routinely applied for lung SBRT was called “kV-Chest_1_” throughout this study.
- **Clinical kV-Chest**_**2**_**-CBCT**
  The preset “kV-Chest_2_” of the recently introduced XVI version 5.0.2, installed on the Elekta VersaHD linac, differed slightly from the previous preset kV-Chest_1_. A bow-tie filter was applied and the mAs was reduced, as thus was the nominal scan dose (CT dose per volume). Acquisition time remained the same (2min); however, beam interruptions were possible in case of DIBH treatment, extending imaging time to 3-4min.
- **kV-MV-CBCT**
  The kV-MV-CBCT imaging technique was set up as a simultaneous 90° kV and 90° MV scan, achieved by dedicated in-house developed synchronization hard- and software. Since the MV-panel was initially not developed for continuous imaging, linac-pulsing and panel-readout had to be triggered alternately. Due to limited readout-speed, one MV-projection could only be acquired with every fifth kV-projection, resulting in 19 MV-projections versus 100 kV-projections for a 90° scan (+ cone-angle of 10°).
  Linac-parameters were adjusted with our automated workflow to achieve low-dose output of 4–5 Monitor Units (MU) distributed equally over the 19 MV-projections, depending on wear of magnetron and electron gun. At the time of this study, the output was 4MU for a 100° rotation. Acquired kV- and MV-volumes were reconstructed separately and combined after appropriate grey-scale matching [13,14,16,17].

**Table 1:** Technical setup of applied imaging presets.

Preset	kV-Chest_1_-CBCT	kV-Chest_2_-CBCT	kV-MV-CBCT
**Output characteristics**	120kVp	120kVp	**kV:** 100kVp
	1mAs (25mA, 40ms) per frame	0.4mAs (20mA, 20ms) per frame	0.1mAs (10mA, 10ms) per frame
			**MV:** 6MV linac (4MU)
**Number of frames**	650	660	**kV:** 100
			**MV:** 19
**Panel position**	off-centered (M)	off-centered (M)	centered (S)
**Filter,**	F0 (empty),	F1 (bow-tie),	**kV:** F0 (empty),
**Collimator (field size)**	M20 (27.67cm x 42.64cm)	M20 (27.67cm x 42.64cm)	S20 (27.67cm x 27.67cm)
			**MV:** 25cm x 25cm
**Rotation speed**	½ rpm	½ rpm	1 rpm
**Acquisition length**	360°	360°	90° + cone angle
			(here: 0° to 100°)
**Acquisition time**	2min	minimum 2min	15s
**Nominal dose (uncorrected)**	16mGy	5mGy	**kV:** 0.02mGy
			**MV:** 4MU

### Determination of beam quality and dose correction factors

All absorbed dose measurements were corrected following the AAPM TG protocol 61 [18] (kV-energy range) and the IAEA TPS 398 [19] (MV-energy range). For both energy ranges, measurements were performed with two differently-calibrated air-filled point-dose flexible cylindrical ionization chambers (Type 31013, 0.3cm^3^ sensitive volumes) and a UNIDOS E Universal dosemeter (all PTW Freiburg GmbH, Freiburg, Germany). The first ionization chamber was calibrated in air kerma for kV-range (50-150kVp), the second ionization chamber was calibrated on absorbed-dose-to-water for 60-Cobalt (MV-range).

The beam qualities and calibration factors for absolute dose measurements in both kV-and MV-energy ranges are determined as follows.

#### KV-energy range: Beam quality and correction factors

Following the AAPM TG61 protocol [18], kV beam qualities for the applied beam setups were characterized by measuring the half-value-layers (HVL) of aluminum and calibration factors were determined accordingly. Similar adaptation of the AAPM TG61 protocol for kV-CBCT was performed by Song et al. [20] in 2008.

**Fig 1:**
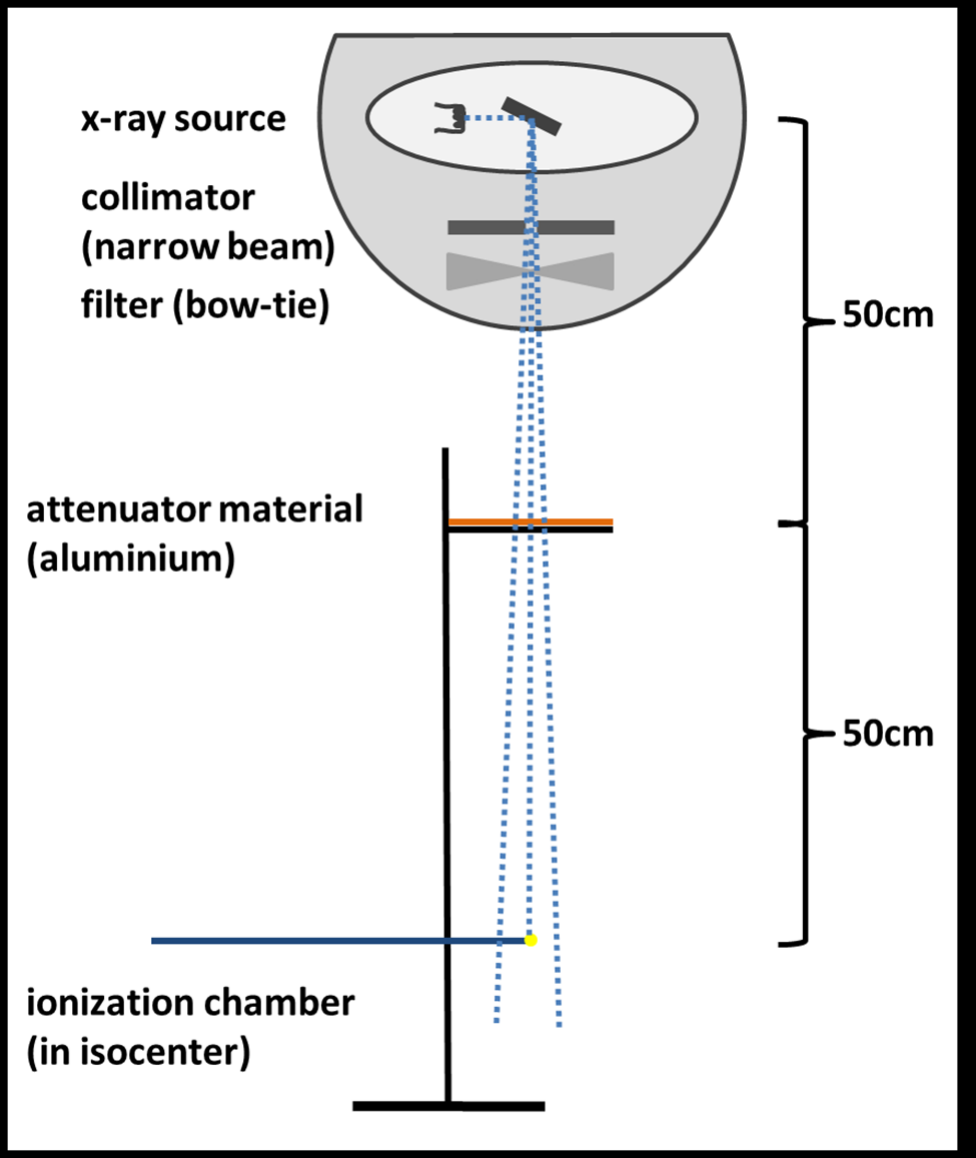
HVL measurement setup.

KV beam quality–determination of HVL: A self-made collimator with field size (5.5x5.5)cm^2^ in the isocenter was used to achieve narrow-beam geometry to minimize scatter effects [21]. The x-ray source was positioned at 0° and the detector was moved out of the field-of-view (FOV). A measurement stand on top of the treatment couch aligned both the ionization chamber and the varying aluminum absorbers with the center of the FOV (see Fig 1). The source to surface distance (SSD) was 100cm for the ionization chamber, the aluminum absorbers were positioned at SSD 50cm. The thickness of the attenuator varied from 0.01–10.31mm, the purity of aluminum was 99.9%.

The HVL was measured for all 3 presets (kV-Chest_1_, kV-Chest_2_ and the kV-contribution of kV-MV), with slight modifications such as static gantry position and adapted number of frames. All HVL measurements were normalized. The preset collimators were replaced with the narrow-beam collimator. To determine the HVL, first, the initial dose output I_0_ was measured free in air (take average value from 4 measurements). Thereafter, the thickness of the aluminum absorbers was increased in different step sizes between 0.01mm and 1.06mm, and dose output was measured. The step sizes around the I_0_/2 region were smallest to increase measurement accuracy around the expected HVL value.

In literature, the HVL is usually calculated under ideal conditions of a monoenergetic x-ray beam, following the Lambert-Beer-Law:

$$I(x)=I_{0}e^{-\mu x}$$ (1)

$${HVL}_{LB}=\frac{\ln2}{\mu }\;,\;\mathrm{for}\;I({HVL}_{LB})=\frac{I_{0}}{2},$$ (2)

where I(x) is the dose output at depth x in a medium, I_0_ is the initial dose output in air, and μ is the attenuation coefficient of the absorber material.

However, in reality, the x-ray tube produces polyenergetic beams (with peak energy kVp). Inherent beam filtration such as aluminum filters is used for beam hardening, i.e. low-energy x-rays are filtered, which would be absorbed by the patient’s body and thus not contribute to imaging but only increase the applied dose to the patient. In 1994, Bjaerngard and Shackford [22] analyzed this beam hardening effect. They suggested the following mathematical description of a second-order polynomial in thickness, to analyze the transmission characteristics in terms of varying attenuation coefficient μ and beam-hardening coefficient η:

$$I(x)=I_{0}e^{-\mu x(1-\eta x)}=I_{0}e^{-\mu x+\mu \eta x^{2}}$$ (3)

$${HVL}_{BS}=\frac{\mu -\sqrt{\mu^{2}-4\mu \eta \ln2}}{2\mu \eta }$$ (4)

KV beam quality–correction factors: After calculating the HVL for each imaging preset following [22], the appropriate correction factors could be determined from the look-up-tables provided in [18], following the dosimetry formalism for tube voltages between 100-300kV:

$$D_{w}=MN_{k}P_{Q,cham}P_{sheath}\;{\left[{\left(\frac{\mu_{en}}{\rho }\right)}_{air}^{water}\right]}_{water}$$ (5)

with *M* = (*M*_*raw*_ − *M*_0_)*P*_*TP*_*P*_*ion*_*P*_*pol*_*P*_*elec*_

The in-phantom formalism converted the air-kerma calibrated ionization chamber reading M [C] to absorbed dose-to-water D_w_ [Gy]. Further explanations about the correction factors N_k_ (air-kerma calibration), P_Q,cham_ (ionization chamber stem correction), P_sheath_ (ionization chamber waterproofing sheath correction), ${\left[{\left(\frac{\mu_{en}}{\rho }\right)}_{air}^{water}\right]}_{water}$ (water-to-air ratio of the mass-energy-absorption coefficients averaged over the incident photon spectrum), P_TP_ (temperature-pressure correction), P_ion_ (ion collection efficiency), P_pol_ (polarity correction) and P_elec_ (electrometer correction) can be found in Ma et al. [18].

#### MV-energy range: Beam quality and correction factors

The determination of MV-energy beam quality differs from the kV beam quality procedure, because the MV beam is already hardened by heavy inherent filtration of target and flattening filter. A convenient method to determine the energy of the MV beam is to measure the percentage depth dose distribution (PDD) and put it in relation to reference data, i.e. 60-Cobalt.

MV beam quality—determination of quality index Q: Following [19], the PDD was measured in a water tank and the beam quality index Q of our clinical 6MV Synergy linac beam was determined. The reference setup was SSD 100cm, field size was 10x10cm^2^ in isocenter, and the PDD was acquired for a depth of 0-30cm in a water tank (IBA blue phantom, IBA Dosimetry, Schwarzenbruck, Germany). The ionization chamber was positioned in the central axis of the beam and corrected for the effective point of measurement.

MV beam quality–correction factors: From the acquired PDD, the percentage dose values of depths 20cm (M_20_) and 10cm (M_10_) can be selected and the beam quality index can be calculated as follows [19]:

$$Q=1.2661\frac{M_{20}}{M_{10}}-0.0595$$ (6)

The absorbed dose-to-water calculation for high energy beams (MV-range) follow the formalism:

$$D_{w}=MN_{D,w}k_{Q}$$ (7)

with *M* = (*M*_*raw*_ − *M*_0_)*P*_*TP*_*P*_*ion*_*P*_*pol*_*P*_*elec*_

The beam-quality conversion factor k_Q_ converts the calibration factor of the reference energy (Cobalt-60) to the energy of beam quality Q, and can be found in the look-up-table provided in IAEA TRS 398 [19]. Further details about the correction factors can be found in literature [19,23].

### Consideration of relative biological effectiveness

The ICRP-60 and ICRP-103 reports [24,25] suggest to keep a uniform radiation weighting factor of w_R_ = 1 for all low-LET-radiations such as x-rays, electrons and gamma-rays of all energies. However, several studies discovered an increase in biological effectiveness with decreasing photon energy [26–28]. These studies are either based on epidemic data of the Hiroshima and Nagasaki atomic bombs or on *in vitro* radio-biological measures, and show significant differences in biological effectiveness for different energies.

In IGRT, special consideration has to be taken of a potentially increased risk of secondary cancer [29–31]. In the review of Nikjoo et al. [28], some studies were presented in which the RBE between low- and high-energy photons were compared. For the biological endpoint of neoplastic cell transformation, determined RBE-values range from 2 to 4. Hill et al. [26] and Borek et al. [32] determined an RBE = 2 for low vs. an RBE = 1 for high photon energies.

In this dosimetry study, a conservative RBE ratio of 2:1 for kV vs. MV biological effectiveness was chosen with regard to secondary cancer risk. MV-dose was then converted to kV-equivalent dose by reducing the MV-dose by one half and denominated Gray-equivalent [GE].

### Dosimetry phantom setup

Dose measurement comparisons between kV-MV-CBCT and other CBCT-presets were performed on two phantoms. First, dose output was measured in several regions of an inhomogeneous thorax phantom, to simulate patient imaging dose exposure. Second, a CT dose index (CTDI) was determined for kV-MV-CBCT and compared to other imaging techniques.

The dose measurements of kV-MV-CBCT were performed separately for kV- and MV-contribution. Since the beam qualities differed, two differently-calibrated ionization chambers had to be used. This splitting allowed separate evaluation of the dose contributions as well as consideration of RBE to convert MV to kV-equivalent dose, making final absorbed kV-MV dose exposure comparable to the clinical kV-only CBCT-presets.

#### Inhomogeneous thorax phantom–patient simulation

The inhomogeneous thorax phantom (model 002LFC, CIRS, Norfolk, VA, USA) consisted of different materials according to the human thorax, i.e. mimicking two lungs and a spinal cord. In our study, a target volume was defined in the lower left lung of the phantom. This tumor-mimicking inlay was to be located in the isocenter (Fig 1(A)). Dose measurements were performed in representative beam positions of the phantom:

1. tumor in lower left lung
2. left lung
3. right lung
4. upper periphery
5. spinal cord
6. body center

To spare the contralateral lung in analogy to a patient imaging procedure, the kV-MV preset was designed to rotate only on the side of the tumor location, i.e. from gantry position 0–100°.

The MV-contribution of the kV-MV-CBCT was additionally simulated with the Oncentra treatment planning system (Elekta Oncentra Masterplan, Elekta AB, Stockholm, Sweden) for a 4MU per 100° arc.

#### CT dose index—QA phantom

Following the quality assurance (QA) protocol of AAPM reports TG179 [4] and TG75 [33], a standard dose value for regular QA was defined for kV-MV-CBCT. Many CTDI determination and dose comparison studies between different CBCT-presets of Elekta and Varian were published in the last years [34–38]. Initially, the CTDI was developed for diagnostic CT, but with the transmission to CBCT and larger field of views, the proper use of ionization chamber had to be reconsidered. Dixon et al. [39] suggested to use a small point-dose ionization chamber measuring the central beam of CBCT, instead of the commonly used 10cm long ionization chamber.

**Fig 2:**
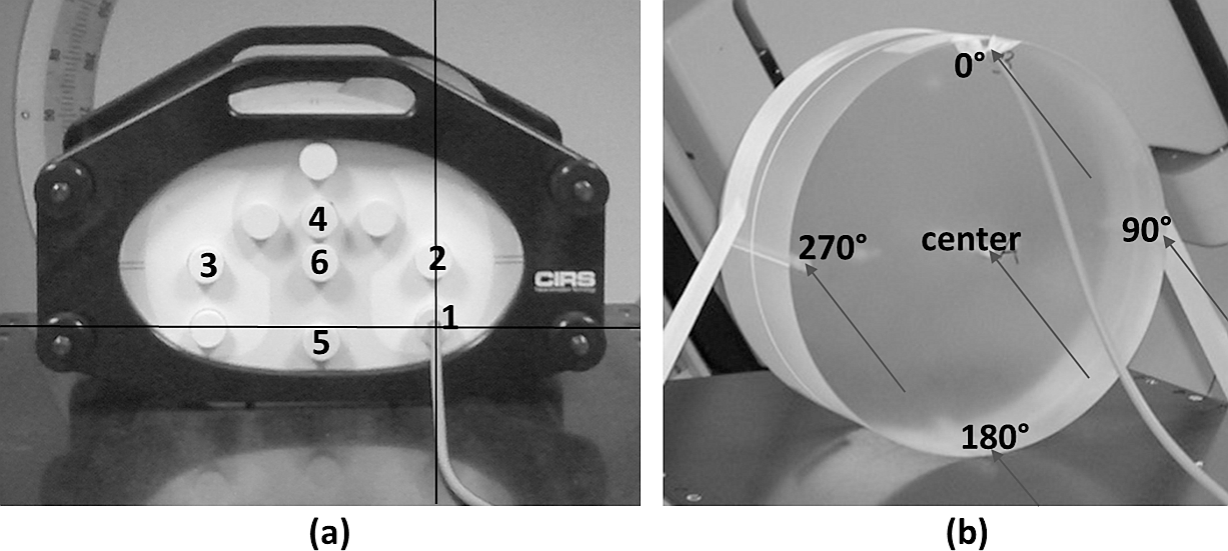
Setup and layout of different measurement positions of (a) inhomogeneous thorax phantom for patient simulation (1: tumor in lower left lung, 2: left lung, 3: right lung, 4: upper periphery, 5: spinal cord, 6: body center), and (b) CT dose body phantom for QA purposes.

In our study, the CTDI measurements were performed in the CT body phantom (T40016, PTW, Freiburg, Germany), made of polymethyl-mathacrylate (PMMA, density ρ = 1.19g/cm^3^), with diameter 32cm and axial length 15cm (Fig 2(B)). The phantom featured five cavities to sequentially insert the ionization chamber: one center and four peripheral positions (at gantry angle 0°, 90°, 180°, 270°). The dose measurements were again performed with the two differently-calibrated (kV- and MV-energy) point-dose ionization chambers. An additional halved dummy plug was placed into the cavities sequentially together with the ionization chamber at axial midpoint (isocenter) of the phantom to prevent the air gap. The weighted CTDI_w_ was calculated as follows [33]:

$${CTDI}_{w}=\frac{1}{3}{CTDI}_{c}+\frac{2}{3}{CTDI}_{p}$$ (8)

with *CTDI*_*c*_ central dose

$$\mathrm{and}\;{CTDI}_{p}=\frac{1}{4}({CTDI}_{p,0{}^{\circ}}+{CTDI}_{p,90{}^{\circ}}+{CTDI}_{p,180{}^{\circ}}+{CTDI}_{p,270{}^{\circ}})$$ (9)

peripheral dose (averaged).

## Results

**Fig 3:**
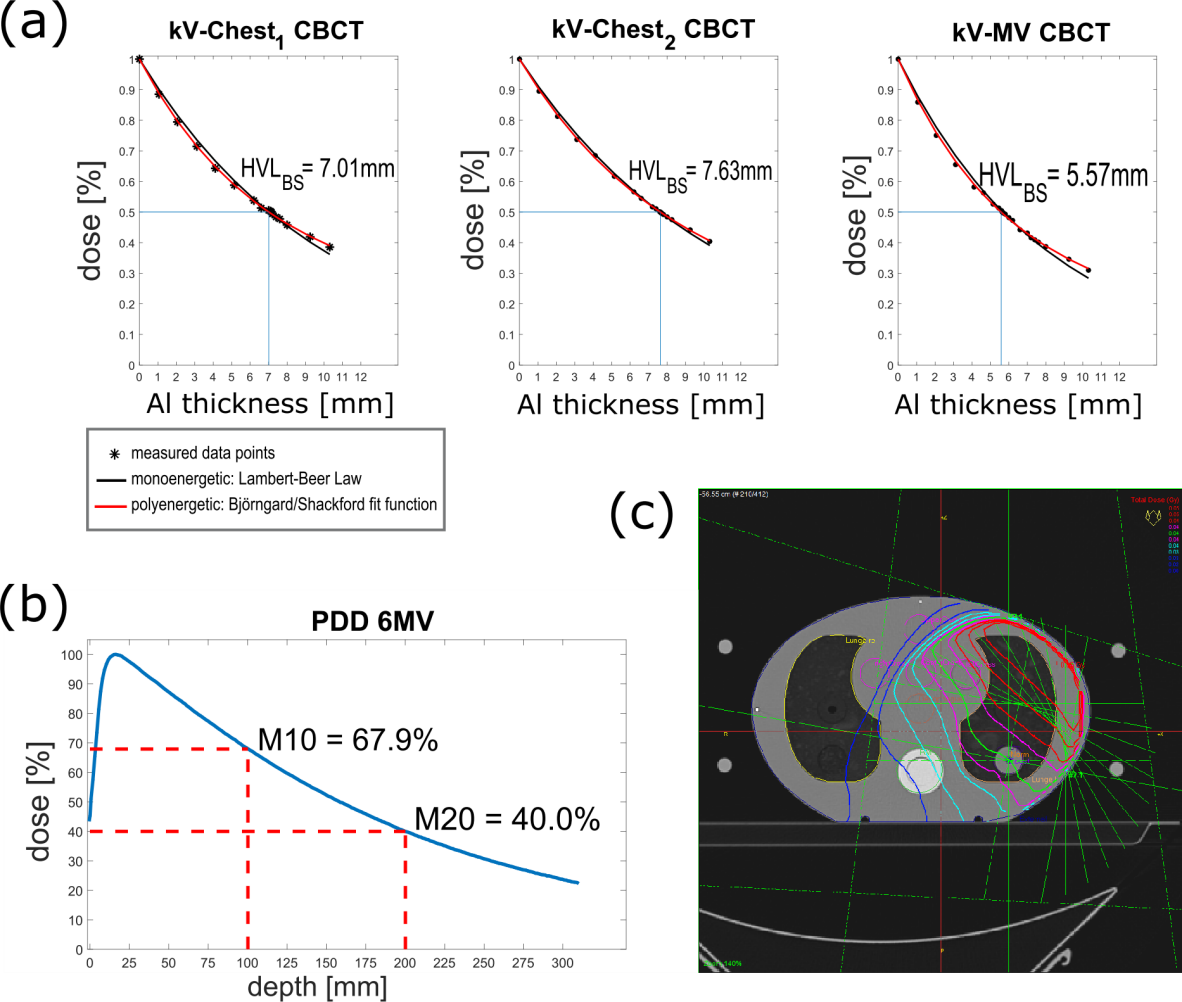
(a) HVL measurement results for all kV-dependent CBCT-presets, (b) PDD for MV beam quality determination, and (c) simulation of 4MU distributed over a 100° gantry arc with the TPS.

Following the AAPM TG61 protocol (kV-energy) [18] for kV-Chest_1_, kV-Chest_2_ and the kV-contribution of kV-MV-CBCT, and the IAEA TRS 398 protocol (MV-energy) [19] for the MV-contribution of kV-MV-CBCT, the beam qualities and corresponding calibration factors for dose measurements were determined and applied (see chapter 2.2 and Table 2). Fig 3(A) shows the HVL measurements and the fit function according to Bjaerngard and Shackford [22] (goodness of fit R^2^ = 0.9988–0.9997), the calculated HVL_BS_ matched with our direct measurement results within a 2% maximum difference and was consistent with measurement values found in literature [18,20,40]. The MV PDD is shown in Fig 3(B).

**Table 2:** Absolute dose correction factors for kV- and MV-energy, based on AAPM TG61 report and IAEA TRS 398, respectively, whereas the values for HVL_BS_ and Q were calculated from the beam quality measurements.

kV-energy correction factors				MV-energy correction factors	
	kV-Chest_1_-CBCT	kV-Chest_2_-CBCT	kV-MV-CBCT		6MV linac
*HVL*_*BS*_	7.01mm Al	7.63mm Al	5.57mm Al	*Q*	0.6858
${\left[{\left(\frac{\mu_{en}}{\rho }\right)}_{air}^{water}\right]}_{water}$	1.0394	1.0417	1.0346	*k*_*Q*_	0,9894
*P*_*Q,cham*_	1.022	1.023	1.019	*N*_*D*,*w*_	1.0·10^8^ Gy/C
*P*_*sheath*_	1	1	1		
*N*_*k*_	8.757·10^7^ Gy/C				

### Inhomogeneous thorax phantom–patient simulation

Column 8 in Table 3 shows the corrected absolute dose output for the various locations (serially numbered) and imaging methods. The dose measurements of kV-MV-CBCT were performed separately (columns 5 and 7), because the dose responses of kV and MV behaved differently. Furthermore, the separate dose contributions could be distinguished and the different RBE could be considered (column 6), enabling proper dose comparison in kV-range. The final absorbed dose of kV-MV-CBCT in column 8 is the sum of the kV-contribution and the kV-equivalent MV-contribution, the unit is [GE].

**Table 3:** Absorbed dose for different presets in inhomogeneous thorax phantom (column 8), ratio between the imaging methods and reference dose output kV-MV (D/D_ref_) (column 9), comparison of MV-contribution dose output between measured and calculated (TPS) absolute dose by percentage difference (column 3–5), and separate contributions of kV- and RBE-corrected MV absorbed dose to kV-MV-CBCT in columns 6+7 (GE: Gray-equivalent with RBE = 2 for kV-dose contribution).

Position	CBCT	calculated (TPS):		measured:			final absorbed dose	*ratio vs*. *kV-MV*
		MV part	*percentaged difference (%)*	MV part	MV part (kV-equivalent)	kV part		
1: tumor- lower left lung	kV-MV	40.40 mGy	*0*.*35*	40.26 mGy	20.13 mGE	0.38 mGy	**20.51 mGE**	*1*.*00*
	kV-Chest_1_						**31.26 mGy**	*1*.*52*
	kV-Chest_2_						**9.10 mGy**	*0*.*44*
2: upper left lung	kV-MV	44.90 mGy	*0*.*45*	45.10 mGy	22.55 mGE	0.33 mGy	22.88 mGE	*1*.*00*
	kV-Chest_1_						32.00 mGy	*1*.*40*
	kV-Chest_2_						9.13 mGy	*0*.*40*
3: right lung	kV-MV	13.40 mGy	*2*.*84*	13.02 mGy	6.51 mGE	0.09 mGy	**6.60 mGE**	*1*.*00*
	kV-Chest_1_						**22.85 mGy**	*3*.*46*
	kV-Chest_2_						**5.22 mGy**	*0*.*79*
4: upper center	kV-MV	37.30 mGy	*1*.*74*	37.95 mGy	18.98 mGE	0.13 mGy	19.10 mGE	*1*.*00*
	kV-Chest_1_						23.06 mGy	*1*.*21*
	kV-Chest_2_						6.21 mGy	*0*.*33*
5: spinal cord	kV-MV	30.00 mGy	*0*.*60*	29.82 mGy	14.91 mGE	0.20 mGy	15.12 mGE	*1*.*00*
	kV-Chest_1_						16.45 mGy	*1*.*09*
	kV-Chest_2_						4.62 mGy	*0*.*31*
6: body center	kV-MV	34.30 mGy	*1*.*55*	34.83 mGy	17.41 mGE	0.16 mGy	17.57 mGE	*1*.*00*
	kV-Chest_1_						21.54 mGy	*1*.*23*
	kV-Chest_2_						5.96 mGy	*0*.*34*

#### Comparison of MV dose measured vs. calculated (TPS)

To demonstrate asymmetric dose distribution of kV-MV-CBCT, the MV-contribution was simulated in the Oncentra TPS. The axial dose distribution of 4MU equally distributed over 100° is shown in Fig 3(C), the calculated MV dose outputs are listed in column 3 of Table 3. In this comparison the RBE was not considered, i.e. the originally measured absorbed dose was shown. The percentage difference between measured and calculated MV dose values with a maximum difference of 2.8% underlined a proper MV dose output also in low-dose mode (column 4 of Table 3).

#### Dose comparison of kV-MV imaging vs. conventional CBCT

Column 9 of Table 3 gives an overview of the ratios between kV-Chest_1_ respective kV-Chest_2_ versus kV-MV-CBCT. Among each other, the two clinical presets kV-Chest_1_ and kV-Chest_2_ differed mainly in the applied nominal tube current (mA) and pulse duration (ms), see Table 1. Furthermore, the bow-tie filter in kV-Chest_2_-CBCT also reduced imaging dose [41]. KV-Chest_1_ resulted in an absolute dose of 31.26mGy in the tumor and 22.85mGy in the contralateral lung. For kV-Chest_2_, these dose values were reduced by a factor of 3–4, resulting in 9.10mGy for the tumor region and 5.22mGy for the organ at risk. Dose output was slightly inhomogeneous distributed around the phantom due to the fact that the phantom was positioned off-centered. The smallest dose was detected in the spinal cord.

KV-MV-CBCT dose outputs lay in between the dose outputs of both kV-presets. Maximum dose of kV-MV-CBCT was 22.88mGE at the left lung position. Minimum dose of 6.60mGE was exposed to the contralateral lung. This was due to asymmetric dose distribution; organs at risk could be spared. At the tumor position, dose output of the kV-MV approach was 20.51mGE, i.e. it was 34% smaller than kV-Chest_1_ and 2.25 times higher than kV-Chest_2_. The largest difference of 3.3 times the dose output of the kV-Chest_2_-CBCT appeared at the position of the spinal cord. Compared to the kV-Chest_1_-CBCT, however, kV-MV dose output was still 8% lower.

### CTDI quality assurance

The CTDI_w_ was calculated following Eq 8. The resulting dose values for each of the five measurement points as well as the final weighted CTDI are listed in Table 4. The MV-contribution of the kV-MV-CBCT dose measurement results were converted to kV-equivalent dose by taking half of the MV dose value. This kV-equivalent MV dose was added to the kV-contribution, resulting in the final absorbed kV-MV-CBCT dose output.

**Table 4:** Determination of weighted CTDI for different CBCT-presets (GE: Gray-equivalent with RBE = 2 for kV-dose contribution) (columns 2, 3 and 5) and ratio between the imaging methods and reference dose output kV-MV (D/D_ref_) (columns 4 and 6).

Position	kV-MV-CBCT	kV-Chest_1_-CBCT	*ratio vs*. *kV-MV*	kV-Chest_2_-CBCT	*ratio vs*. *kV-MV*
**CTDI**_**c**_	14.70 mGE	9.68 mGy	*0*.*66*	3.42 mGy	*0*.*23*
**CTDI**_**p, 0°**_	19.83 mGE	20.52 mGy	*1*.*03*	6.41 mGy	*0*.*32*
**CTDI**_**p, 90°**_	16.72 mGE	20.24 mGy	*1*.*21*	6.00 mGy	*0*.*36*
**CTDI**_**p, 180°**_	5.66 mGE	20.61 mGy	*3*.*64*	5.53 mGy	*0*.*98*
**CTDI**_**p, 270°**_	5.74 mGE	22.27 mGy	*3*.*88*	6.74 mGy	*1*.*17*
**CTDI**_**w**_	**11.99 mGE**	**17.16 mGy**	*1*.*43*	**5.25 mGy**	*0*.*44*

#### Dose comparison of kV-MV imaging vs. conventional CBCT

Among each other, the clinical presets kV-Chest_1_ and kV-Chest_2_ differed in nominal dose output about a factor of 3.2, due to different tube current per pulse duration (mAs) and different filters (see Table 1), which transmits to the different dose output. This difference is also seen in the comparison of CTDI_w_: the measured weighted CT dose index for kV-Chest_1_ is 17.16mGy, and 5.25mGy for kV-Chest_2_. The kV-MV weighted CT dose index was in between both clinical presets, with CTDI_w_ = 11.99mGE.

## Discussion

While currently standard clinical kV-CBCT image acquisition for lung tumor position verification takes about 3-4min in repeated breath-hold, our in-house developed and recently fully established automatic ultrafast combined kV-MV-CBCT method enables imaging within only one single breath-hold of 15s [16,17], providing promising approach for DIBH-patients suffering from lung cancer [8].

For clinical implementation of a novel imaging technique, feasibility has to be evaluated regarding fulfillment of sufficient image quality for registration within 1mm and small dose exposure compared to prescribed dose. Excellent registration accuracy of combined kV-MV-CBCT was recently reported [17]. A remaining issue, however, is the dose exposure to the patient. The MV-contribution of kV-MV-CBCT could be a critical dosimetric issue and therefore merits comparison with standard kV-CBCT.

The kV-MV dose output in this study was composed as a sum of the kV-contribution, and the MV-contribution converted into kV-equivalent dose by considering changes in RBE between both kV- and MV-energies. Following recommendations in literature [26,32], a conservative RBE of 2 vs. 1 was chosen for kV vs. MV dose.

A hallmark of kV-MV-CBCT is its asymmetric dose distribution, due to the limited gantry angle rotation of 90°. The maximum dose was in the left lung where the tumor was located and the kV- and MV-contributions overlapped. Healthy tissue such as the contralateral lung could therefore be spared; kV-MV dose in the healthy right lung was thus 2/3 lower than the clinical preset kV-Chest_1_. Compared to latest available kV-Chest_2_, the kV-MV-CBCT dose is up to 3.3 times higher, whereas the dose value in the contralateral lung was almost the same, due to homogeneous distribution of kV-Chest_2_.

In 2015, Alaei and Spezi [42] published a detailed review of more than 500 publications on both kV and MV-CBCT imaging dose. Most of the presented studies performed dose measurements with the clinical kV-CBCT presets provided by the manufacturers. They either measured CTDI [20,34,43], or tried to estimate imaging dose to the patient by phantom measurements [44,45] or Monte Carlo simulations [37,46]. However, previous studies did not consider the different RBE of kV imaging [43], neither in their dose calculations, nor in their risk analysis. Not only physical dose but also its biological relevance should therefore be considered in any comparison. The trade-off between benefits of frequent imaging and its downside, additional dose to non-target tissues, is currently discussed [43,47,48]. All in [42] reviewed fractional dose values vary strongly due to different methods and phantoms, but common kV dose values are reported to be between 10-50mGy for internal organs and up to 70mGy for skin. For linac-based MV-CBCT, doses of 100-150mGy per fraction are typical if the 6MV therapeutic beam is used, and is therefore usually accounted for in treatment planning [6,7]. The MV-CT installed on a Hi-Art Tomotherapy unit however uses 3.5MV for imaging and results in the range of 20mGy for CTDI_w_ [49]. All these values however do not consider different RBE for kV- and MV-energy range. In 2015, Liu et al. [50] published a theoretical approach to combine kV- and MV-CBCT imaging focusing on image quality only with their fractional imaging doses of 0.5-2Gy, making a clinical implementation impossible at this stage of research. The non-RBE corrected dose output of our novel kV-MV-CBCT implementation, however, already produced an acceptable range from 13.11–45.43mGy in inhomogeneous thorax phantom measurements. In case of different wear of magnetron and electron gun, an MV output of 5MU would lead to a dose output between ~16-57mGy. In our approach, exceeding 5MU per 90° arc (+ cone-angle) would not be possible because of hard-coded interlock in our automated kV-MV workflow [14,16].

Therefore it can be concluded that not only the dose output results of our clinical CBCT-presets kV-Chest_1_ and kV-Chest_2_ are in close agreement with the above-mentioned literature [42], but also kV-MV-CBCT. The MV-contribution is relatively small compared to MV-CBCT dose outputs in literature [6,42] or standard EPID verifications (~4MU) and does not have to be considered in treatment planning, because we triggered the MV-output to low dose and our imaging time is reduced to only 15s [14,16]. Furthermore, kV-MV could reduce dose exposure to sensitive patient skin due to the MV-build-up effect.

Eventually, the most important aspect of the dosimetric properties of kV-MV-CBCT is that the determined imaging doses of approximately 22mGE/ 45mGy remain negligible compared to the prescribed dose, particularly for SBRT [43].

## Conclusions

This comparison study demonstrated that the dosimetric properties of kV-MV-CBCT were in agreement with common clinically established imaging techniques, whereas biologically effective exposure to healthy tissue was reduced. Together with the previously published report on registration accuracy with kV-MV-CBCT [17], this dosimetric study demonstrates that kV-MV-CBCT fulfills all necessary requirements for imminent clinical implementation.

## References

[1] Goldsmith C, Gaya A. Stereotactic ablative body radiotherapy (SABR) for primary and secondary lung tumours. Cancer Imaging. 2012;12: 351–360. doi: 10.1102/1470-7330.2012.9015 23023165 10.1102/1470-7330.2012.9015 PMC3460596

[2] Timmerman RD, Kavanagh BD, Cho LC, Papiez L, Xing L. Stereotactic body radiation therapy in multiple organ sites. J Clin Oncol. 2007 pp. 947–952. doi: 10.1200/JCO.2006.09.7469 17350943 10.1200/JCO.2006.09.7469

[3] Jaffray D, Siewerdsen J. Flat-panel cone-beam computed tomography for image-guided radiation therapy. Int J Radiat Oncol Biol Phys. 2002;53: 1337–1349. 12128137 10.1016/s0360-3016(02)02884-5

[4] Bissonnette J-P, Balter PA, Dong L, Langen KM, Lovelock DM, Moseley DJ, et al Quality assurance for image-guided radiation therapy utilizing CT-based technologies: A report of the AAPM TG-179. Med Phys. 2012;39: 1946–1963. doi: 10.1118/1.3690466 22482616 10.1118/1.3690466

[5] Boda-Heggemann J, Lohr F, Wenz F, Flentje M, Guckenberger M. kV cone-beam CT-based IGRT: a clinical review. Strahlenther Onkol. 2011;187: 284–91. doi: 10.1007/s00066-011-2236-4 21533757 10.1007/s00066-011-2236-4

[6] Chen J, Morin O, Aubin M, Bucci MK, Chuang CF, Pouliot J. Dose-guided radiation therapy with megavoltage cone-beam CT. Br J Radiol. 2006;79 Spec No: S87–98. 16980688 10.1259/bjr/60612178

[7] Pouliot J, Bani-Hashemi A, Svatos M, Ghelmansarai F, Mitschke M, Aubin M, et al Low-dose megavoltage cone-beam CT for radiation therapy. Int J Radiat Oncol Biol Phys. 2005;61: 552–560. 15736320 10.1016/j.ijrobp.2004.10.011

[8] Boda-Heggemann J, Knopf A-C, Simeonova A, Wertz H, Stieler F, Jahnke A, et al DIBH (Deep Inspiratory Breath Hold)-based radiotherapy–a clinical review. Int J Radiat Oncol Biol Phys. 2015;94: 478–492. doi: 10.1016/j.ijrobp.2015.11.049 26867877 10.1016/j.ijrobp.2015.11.049

[9] Boda-Heggemann J, Jahnke A, Jahnke L, Simeonova A, Mai SK, Wertz H, et al Breath-Hold Cone Beam CT (CBCT): Improved Image Quality With “Stop-and-Go” Breath Hold-Only Acquisition Versus Repetitive Breath Hold During Continuous Rotation. Int J Radiat Oncol Biol Phys. 2014;90: S826.

[10] Simeonova AO, Jahnke A, Jahnke L, Siebenlist K, Stieler F, Mai S, et al Automatically Gated CBCT-Controlled Fast Breath-Hold SBRT Is Dosimetrically Robust and Facilitates Precision Treatments for Patients With Lung Cancer. Int J Radiat Oncol Biol Phys. 2014;90: S891.

[11] Navarria P, Ascolese AM, Mancosu P, Alongi F, Clerici E, Tozzi A, et al Volumetric modulated arc therapy with flattening filter free (FFF) beams for stereotactic body radiation therapy (SBRT) in patients with medically inoperable early stage non small cell lung cancer (NSCLC). Radiother Oncol. 2013;107: 414–418. doi: 10.1016/j.radonc.2013.04.016 23725859 10.1016/j.radonc.2013.04.016

[12] Boda-Heggemann J, Mai S, Fleckenstein J, Siebenlist K, Simeonova A, Ehmann M, et al Flattening-filter-free intensity modulated breath-hold image-guided SABR (Stereotactic ABlative Radiotherapy) can be applied in a 15-min treatment slot. Radiother Oncol. 2013;109: 505–509. doi: 10.1016/j.radonc.2013.09.014 24128805 10.1016/j.radonc.2013.09.014

[13] Blessing M, Stsepankou D, Wertz H, Arns A, Lohr F, Hesser J, et al Breath-hold target localization with simultaneous kilovoltage/megavoltage cone-beam computed tomography and fast reconstruction. Int J Radiat Oncol Biol Phys. 2010;78: 1219–26. doi: 10.1016/j.ijrobp.2010.01.030 20554124 10.1016/j.ijrobp.2010.01.030

[14] Wertz H, Stsepankou D, Blessing M, Rossi M, Knox C, Brown K, et al Fast kilovoltage/megavoltage (kVMV) breathhold cone-beam CT for image-guided radiotherapy of lung cancer. Phys Med Biol. 2010;55: 4203–17. doi: 10.1088/0031-9155/55/15/001 20616405 10.1088/0031-9155/55/15/001

[15] Yin F-F, Guan H, Lu W. A technique for on-board CT reconstruction using both kilovoltage and megavoltage beam projections for 3D treatment verification. Med Phys. 2005;32: 2819–2826. doi: 10.1118/1.1997307 16266096 10.1118/1.1997307

[16] Blessing M, Arns A, Wertz H, Stsepankou D, Boda-Heggemann J, Lohr F, et al Image Guided Radiation Therapy Using Ultrafast kV-MV CBCT: End-to-End Test Results of the Finalized Implementation. Int J Radiat Oncol Biol Phys. 2014;90: S828–S829.

[17] Arns A, Blessing M, Fleckenstein J, Stsepankou D, Boda-Heggemann J, Simeonova-Chergou A, et al Towards clinical implementation of ultrafast combined kV-MV CBCT for IGRT of lung cancer. Strahlenther Onkol. 2016;192: 312–321. doi: 10.1007/s00066-016-0947-2 26864049 10.1007/s00066-016-0947-2

[18] Ma C-M, Coffey CW, DeWerd LA, Liu C, Nath R, Seltzer SM, et al AAPM protocol for 40–300 kV x-ray beam dosimetry in radiotherapy and radiobiology. Med Phys. 2001;28: 868–93. doi: 10.1118/1.1374247 11439485 10.1118/1.1374247

[19] IAEA. Absorbed Dose Determination in External Beam Radiotherapy: An International Code of Practice for Dosimetry Based on Standards of Absorbed Dose to Water, Technical Reports Series No. 398. Vienna; 2000.

[20] Song WY, Kamath S, Ozawa S, Al Ani S, Chvetsov A, Bhandare N, et al A dose comparison study between XVI and OBI CBCT systems. Med Phys. 2008;35: 480–6. doi: 10.1118/1.2825619 18383668 10.1118/1.2825619

[21] Trout D, Kelley JP. Beam Quality Measurements in Diagnostic Roentgenology. Am J Roentgenol. 1971;112: 622–627. 10.2214/ajr.112.3.622 5570376

[22] Bjaerngard BE, Shackford H. Attenuation in high-energy x-ray beams. Med Phys. 1994;21: 1069–73. doi: 10.1118/1.597349 7968838 10.1118/1.597349

[23] Almond PR, Biggs PJ, Coursey BM, Hanson WF, Huq MS, Nath R, et al AAPM’s TG-51 protocol for clinical reference dosimetry of high-energy photon and electron beams. Med Phys. 1999;26: 1847–1870. doi: 10.1118/1.598691 10505874 10.1118/1.598691

[24] ICRP. 1990 Recommendation of the International Commission of Radiological Protection. ICRP Publ 60 Ann ICRP. 1991;21.

[25] ICRP. 2007 Recommendation of the International Commission of Radiological Protection. ICRP Publ 103 Annu ICRP. 2007;37.

[26] Hill MA. The variation in biological effectiveness of X-rays and gamma rays with energy. Radiat Prot Dosimetry. 2004;112: 471–81. doi: 10.1093/rpd/nch091 15623881 10.1093/rpd/nch091

[27] Hunter N, Muirhead CR. Review of relative biological effectiveness dependence on linear energy transfer for low-LET radiations. J Radiol Prot. 2009;29: 5–21. doi: 10.1088/0952-4746/29/1/R01 19225189 10.1088/0952-4746/29/1/R01

[28] Nikjoo H, Lindborg L. RBE of low energy electrons and photons. Phys Med Biol. 2010;55: R65–109. doi: 10.1088/0031-9155/55/10/R01 20427859 10.1088/0031-9155/55/10/R01

[29] Green S, Jones B. Second cancer risk. Br J Radiol. 2005 pp. 469–470. 10.1259/bjr/89346355 15845947

[30] Guckenberger M, Krieger T, Richter A, Baier K, Wilbert J, Sweeney RA, et al Potential of image-guidance, gating and real-time tracking to improve accuracy in pulmonary stereotactic body radiotherapy. Radiother Oncol. 2009;91: 288–295. doi: 10.1016/j.radonc.2008.08.010 18835650 10.1016/j.radonc.2008.08.010

[31] Kirkby C, Ghasroddashti E, Poirier Y, Tambasco M, Stewart RD. RBE of kV CBCT radiation determined by Monte Carlo DNA damage simulations. Phys Med Biol. 2013;58: 5693–704. doi: 10.1088/0031-9155/58/16/5693 23899567 10.1088/0031-9155/58/16/5693

[32] Borek C, Hall EJ, Zaider M. X rays may be twice as potent as γ rays for malignant transformation at low doses. Nat. 1983;301: 156–158. 10.1038/301156a0 6823292

[33] Murphy MJ, Balter J, Balter S, BenComo JA, Das IJ, Jiang SB, et al The management of imaging dose during image-guided radiotherapy: Report of the AAPM Task Group 75. Med Phys. 2007;34: 4041–63. doi: 10.1118/1.2775667 17985650 10.1118/1.2775667

[34] Amer A, Marchant T, Sykes J, Czaika J, Moore C. Imaging doses from the Elekta Synergy X-ray cone beam CT system. Br J Radiol. 2007;954: 476–82. 10.1259/bjr/80446730 17684077

[35] Cheng HCY, Wu VWC, Liu ESF, Kwong DLW. Evaluation of radiation dose and image quality for the Varian cone beam computed tomography system. Int J Radiat Oncol Biol Phys. 2011;80: 291–300. doi: 10.1016/j.ijrobp.2010.06.014 20932676 10.1016/j.ijrobp.2010.06.014

[36] Hyer DE, Hintenlang DE. Estimation of organ doses from kilovoltage cone-beam CT imaging used during radiotherapy patient position verification. Med Phys. 2010;37: 4620–6. doi: 10.1118/1.3476459 20964180 10.1118/1.3476459

[37] Kim S, Yoo S, Yin F-F, Samei E, Yoshizumi T. Kilovoltage cone-beam CT: Comparative dose and image quality evaluations in partial and full-angle scan protocols. Med Phys. 2010;37: 3648–59. doi: 10.1118/1.3438478 20831072 10.1118/1.3438478

[38] Kamath S, Song W, Chvetsov A, Ozawa S. An image quality comparison study between XVI and OBI CBCT systems. J Appl Clin Med Phys. 2011;12: 376–390. 10.1120/jacmp.v12i2.3435 PMC5718664 21587192

[39] Dixon RL. A new look at CT dose measurement: Beyond CTDI. Med Phys. 2003;30: 1272–80. doi: 10.1118/1.1576952 12852553 10.1118/1.1576952

[40] Hyer D, Serago C, Kim S. An organ and effective dose study of XVI and OBI cone-beam CT systems. J Appl Clin Med Phys. 2010;11: 181–197. 10.1120/jacmp.v11i2.3183 PMC5719945 20592702

[41] Mail N, Moseley DJ, Siewerdsen JH, Jaffray D a. The influence of bowtie filtration on cone-beam CT image quality. Med Phys. 2009;36: 22–32. 10.1118/1.3017470 19235370

[42] Alaei P, Spezi E. Imaging dose from cone beam computed tomography in radiation therapy. Phys Medica. 2015;31: 647–658. 10.1016/j.ejmp.2015.06.003 26148865

[43] Sykes JR, Lindsay R, Iball G, Thwaites DI. Dosimetry of CBCT: methods, doses and clinical consequences. J Phys Conf Ser. 2013;444: 12017.

[44] Ding GX, Coffey CW. Dosimetric evaluation of the OneDose MOSFET for measuring kilovoltage imaging dose from image-guided radiotherapy procedures. Med Phys. 2010;37: 4880–5. 10.1118/1.3483099 20964206

[45] Islam MK, Purdie TG, Norrlinger BD, Alasti H, Moseley DJ, Sharpe MB, et al Patient dose from kilovoltage cone beam computed tomography imaging in radiation therapy. Med Phys. 2006;33: 1573–82. doi: 10.1118/1.2198169 16872065 10.1118/1.2198169

[46] Ding GX, Duggan DM, Coffey CW. Accurate patient dosimetry of kilovoltage cone-beam CT in radiation therapy. Med Phys. 2008;35: 1135–44. doi: 10.1118/1.2839096 18404948 10.1118/1.2839096

[47] Spezi E, Downes P, Jarvis R, Radu E, Staffurth J. Patient-specific three-dimensional concomitant dose from cone beam computed tomography exposure in image-guided radiotherapy. Int J Radiat Oncol Biol Phys. 2012;83: 419–426. doi: 10.1016/j.ijrobp.2011.06.1972 22027261 10.1016/j.ijrobp.2011.06.1972

[48] Doerr W, Herrmann T. Second tumors after oncologic treatment. Strahlenther Onkol. 2008;184: 67–72. doi: 10.1007/s00066-008-1807-5 18259697 10.1007/s00066-008-1807-5

[49] Liu M, Wang Y, Liao X. Computed Tomography Dose Index Measurement Voltage Helical CT For Hi-ART Megavoltage Helical CT. Radiat Prot Dosimetry. 2015;12 8: 1–5. 10.1093/rpd/ncv393 26656075

[50] Liu L, Antonuk LE, El-Mohri Y, Zhao Q, Jiang H. Theoretical investigation of the design and performance of a dual energy (kV and MV) radiotherapy imager. Med Phys. 2015;42: 2072–2084. doi: 10.1118/1.4915120 25832097 10.1118/1.4915120

